# Officers of Health in the French Provinces

**Published:** 1839-04

**Authors:** 


### "OFFICERS OF HEALTH" IN THE FRENCH PROVINCES.

As long as quacks and conjurors are permitted by law to impose upon public credulity, a manifest injustice will be suffered by regular practitioners. The labour, the time, and the expenses, which these undergo to qualify themselves to engage the confidence of the sick, are all rendered vain; inasmuch as the quack who starts before him professes to cure all manner of diseases *gratis,* i. e., without any expense to himself of labour, time, or consideration.

*Officers of health,* as they are called, have for a long time been established in the French provinces, and permitted to practise by virtue of a diploma given upon so slight an examination as amounts merely to an attestation of their incapacity. M. Sampicci has undertaken to expose this abuse; and, after stating the disadvantage under which a young and properly qualified practitioner would settle in one of the provincial towns, owing to the formal permission of those who were unqualified, he gives specimens of the kind of examinations to which the *officers* are subjected.

*First Candidate* (*Licensed.*)

*Questions by the Examiners.* What is opium?—Candidate. It is a juice.

Ex. A juice of what?—C. Of an Indian tree.

Ex. And of what family of trees?—C. (Hearing somebody whisper *pap.*) Of the family of the *Popes!*

Ex. Of which of them?—C. (Hearing another whisper papa*ver.*) Of the *green* ones. ("Des verts.")

*Second Candidate* (*Licensed.*)

Ex. How do you recognize a pneumonia?—C. By auscultation and percussion.

Ex. And what do you gain by that?—C. We percuss and we listen.

Ex. And after all what do you find?—C. We find a pneumonia.

*Third Candidate.*

Ex. Prescribe a diuretic draught.—C. Common water, 1 pound; powder of squills, 1 grain.

Ex. But this is not a draught; it is a tisane which you have made, and moreover an homœopathic tisane.—C. We can diminish the dose (replied the candidate, who did not understand what was said.) However, this candidate was admitted, only cautioned (ironically no doubt) to be circumspect in his practice.

*Fourth Candidate.*

Ex. What disease most frequently succeeds to scarlatina?—C. Every disease may succeed to it.

Ex. But is there not one in particular more liable to do so in this case?—C. (Hearing some one whisper anasarca replied),—Nasarca.

Ex. What is that?—C. It is a long time since I dissected, sir.

Ex. Tell me what is an eruptive disease?—C. It is an irritation of inflammation.

*Fifth Candidate.*

Ex. Are there not some hollow muscles?—name some of them.—C. There are the thyroid and the kidneys, (a female accoucheur apprentice here whispered *la matrice*;)—the candidate, who supposed that she was laughing at him, turned round and said aloud, "Madame, you may speak for yourself."

*Bulletin Général de Thérapeutique. Oct.* 15 *et* 30, 1838.

---

MEDICAL FEES IN AMERICA.

